# The Macro-Autophagy-Related Protein Beclin-1 Immunohistochemical Expression Correlates With Tumor Cell Type and Clinical Behavior of Uveal Melanoma

**DOI:** 10.3389/fonc.2020.589849

**Published:** 2020-11-20

**Authors:** Giuseppe Broggi, Antonio Ieni, Daniela Russo, Silvia Varricchio, Lidia Puzzo, Andrea Russo, Michele Reibaldi, Antonio Longo, Giovanni Tuccari, Stefania Staibano, Rosario Caltabiano

**Affiliations:** ^1^ Section of Anatomic Pathology, Department Gian Filippo Ingrassia, University of Catania, Catania, Italy; ^2^ Section of Pathology, Department of Human Pathology in Adult and Developmental Age “Gaetano Barresi”, University of Messina, Messina, Italy; ^3^ Pathology Unit, Department of Advanced Biomedical Sciences, University of Naples Federico II, Naples, Italy; ^4^ Department of Ophthalmology, University of Catania, Catania, Italy; ^5^ Department of Surgical Science, Eye Clinic, University of Torino, Torino, Italy

**Keywords:** uveal melanoma, autophagy, immunohistochemistry, prognosis, Beclin-1

## Abstract

Uveal melanoma, in spite of its rarity, represents the most common primitive intraocular malignant neoplasm of the adults; it affects choroid, ciliary bodied and iris and remains clinically silent for a long time, being accidentally discovered by routine ophthalmic exams. Prognosis of uveal melanoma is poor and frequently characterized by liver metastases, within 10–15 years from diagnosis. Autophagy is a multi-step catabolic process by which cells remove damaged organelles and proteins and recycle nutrients. It has been hypothesized that in early stages of tumorigenesis autophagy has a tumor suppressor role while, in more advanced stages, it may represent a survival mechanism of neoplastic cells in response to stress. Several proteins related to autophagy cascade have been investigated in numerous subtypes of human cancer, with overall controversal results. In this paper we studied the immunohistochemical expression of 3 autophagy related proteins (Beclin-1, p62 and ATG7) in a cohort of 85 primary uveal melanoma treated by primary enucleation (39 with metastasis and 46 non metastatic) and correlated their expression with clinico-pathological parameters and blood vascular microvessel density, in order to investigate the potential prognostic role of autophagy in this rare neoplasm. We found that high immunohistochemical levels of Beclin-1 correlated with a lower risk of metastasis and higher disease-free survival times, indicating a positive prognostic role for Beclin-1 in uveal melanoma. No statistically significative differences regarding the expression of ATG7 and p62 between metastatic and non metastatic patients was detected.

## Introduction

Although uveal melanoma (UM) is traditionally considered a rare tumor, it is the most frequent primitive intraocular malignancy of the adults and the second most frequent melanoma not associated with epithelial structures (1–3). UM is extremely rare in children in whom it recognizes a congenital origin and is often diagnosed in advanced stages of disease with extraocular spreading (4). Choroid is the most common intraocular site involved, followed by ciliary bodies and, less frequently, iris (3). The occurrence of uveal melanoma is 200 times higher in Caucasians, Fitzpatrick phototypes I-II (scores 1 to 13), than in the black population (5, 6). Nevertheless, the ultimate role of UV-B and UV-A exposure as a risk factor for uveal melanoma is still to be fully clarified. Recent reports evidenced that the use of sun-tanning devices is a well-recognized risk factor for uveal melanoma, as opposed to sunlight exposure, hypothesizing that this phenomenon could be correlated to the inability of artificial tanning to increase systemic level of vitamin D3 (7). Presence of choroidal nevus, syndromic/congenital diseases such as cutaneous dysplastic nevus syndrome, oculo-dermal melanocytosis (Ota nevus) and type 1 neurofibromatosis, have traditionally been included among risk factors associated with the onset of UM (8). Sudden retinal detachment is the most frequent presentation of disease; however, this tumor is often clinically silent for a long time, and it is frequently accidentally discovered by routine ophthalmic exams (9, 10). Although several steps have been made in the knowledge and treatment of this neoplasm, the prognosis of UM remains poor with about half of patient developing distant metastases, especially in the liver, within 10-15 years from primary enucleation (10).

To date, the peculiar interplay between the molecular routes that underlie the clinical behavior of UM has still to be fully clarified (11). Deregulation of cell death programs is a hallmark of cancer development, progression, and resistance to therapies (12, 13). A negative regulation of pro-apoptotic molecules by oncogenic signaling (14) and the involvement of several anti-apoptotic proteins involved in the regulation of extrinsic and intrinsic apoptotic pathways have been largely reported in melanoma (15, 16). Recently, also non-apoptotic, autophagy-dependent cell death pathways necroptosis, ferroptosis, pyroptosis, and parthanatos have been found involved in skin melanoma cell response to therapy. Melanoma cells use autophagy to counteract drug activity and tumor microenvironment drug-induced changes, conditioning clinical outcome and resistance to target therapy and immunotherapy (17, 18).

Autophagy is a catabolic process through which damaged organelles and proteins are removed and nutrients recycled by cells (19, 20). During the initiation and development of tumors, autophagy is likely to mainly play a tumor suppressor role while, in well-established cancers, it may constitute a survival mechanism in response to stress (21). Up to now, its role in cancer remains controversial, particularly for its relationship with prognosis. Three main dynamic autophagy pathways exist: chaperone-mediated autophagy (CMA), microautophagy, and macroautophagy (22). CMA is a selective multistep autophagy pathway that modulates the turnover of soluble cytosolic proteins, delivering them directly to lysosomes for degradation, without formation of vesicles (23). Microautophagy leads to the degradation of intracellular proteins and organelles directly engulfed by lysosomes or endosomes. The extent and role of microautophagy in mammalian cells have been yet poorly understood (24). Macroautophagy is a multistep process characterized by the formation of autophagosomes (double-membraned coated vesicles), that fuse with the membrane of lysosome, that degrade and recycle their contents (25). The macroautophagic process is regulated by six autophagy-related (ATG) protein classes and/or complexes (26). Autophagy cannot be detected in routine hematoxylin and eosin-stained and formalin-fixed and paraffin-embedded tissues. Biomarkers detection by IHC constitutes at present the best way to examine this process on routine tissue sections (27). Many of more than 30 autophagy-related genes (ATG) encoded proteins in human cells are detectable by immunohistochemistry (28, 29).

In this study, we choose to immunohistochemically investigate the expression of three proteins involved in the macroautophagic step of the autophagy cascade, Beclin-1, the autophagy-related gene 7 (ATG7), and p62, in a cohort of 62 patients affected by UM at different clinical stage.

BECN1 (Beclin-1) is the mammalian ortholog of yeast autophagy-related gene 6 (ATG6), which induces autophagosome formation. The human Beclin-1 gene is located on chromosome 17q21, and in the early stages of autophagy interacts with various cofactors forming a complex necessary for the recruitment of other ATG proteins and for the progression of the autophagic process (29–32). The evidence that Beclin-1 expression is monoallelicaly suppressed in some human breast, ovarian and prostatic cancer cell lines, suggests that it acts as a tumor suppressor gene (30–32). A decreased expression of Beclin-1 at mRNA and protein levels has been found in human brain tissues (33) and in estrogen receptor-negative, HER2-positive breast cancer, in which the reduced BECN1mRNA expression has been found associated with poor prognosis (34). In addition, upstream positive regulators of Beclin-1, such as UV radiation and Bax interacting factor-1 (Bif-1), have been found downregulated in several types of human cancers (35, 36) and an increased tumor incidence has been reported in Beclin1 heterozygous mice.

All these evidences support the tumor-suppressive function of macroautophagy (37). By converse, macroautophagy can be induced by cancer therapy, and in this eveniences, it has been found to support tumor cell survival, suggesting the gain of oncogenic function (38, 39). The role of macroautophagy in tumorigenesis, then, looks very complex and controversial, with sensible variation among different tumor types and/or different tumor stage (40). ATG7, located on chromosome 3p25.3, encodes an E1-like activating enzyme essential for cytoplasmic to vacuole transport (41). The encoded protein also has modulating function on p53-dependent cell cycle pathways during sustained metabolic stress phases (41). The expression of ATG7 has been recently found to correlate with resistance to anticancer drugs (42). p62 acts as an adaptor protein, through the binding of ubiquitylated protein aggregates and the deliver to the autophagosomes (32, 43, 44). A p62-dependent response triggered to counteract oxidative stress has been reported in melanoma cells exposed to ultraviolet A (UVA) (45).

The aim of this paper is to investigate any relationship among the expression of these three macroautophagy-related proteins and clinical-pathological parameters and behavior of UM.

## Materials and Methods

Clinical data and histological samples of 85 patients affected by primary UM, who underwent primary enucleation at the Eye Clinic of both University of Catania and University of Naples “Federico II” during the ten years up to October 2019, were retrospectively analyzed. All cases were not eligible for non-surgical procedures, such as plaque brachytherapy or proton beam radiotherapy. The research protocol for this retrospective study was approved by our Institutional Medical Ethical Committee (according to the ethical guidelines of the Declaration of Helsinki). Paraffin-embedded tissue samples from each case were selected from the surgical pathology archive of the Sections of Anatomic Pathology, Department G.F. Ingrassia, University of Catania, and Department of Advanced Biomedical Sciences, University of Naples “Federico II”. The following exclusion criteria were used for the selection of cases: i) paraffin blocks in which there was not enough tissue to get additional slides for immunohistochemistry; ii) representative neoplastic tissue was absent; iii) necrosis was the major component of the tumor blocks; iv) the tumor underwent preoperative radiotherapy. Five or more sections were obtained from each paraffin block. Five pathologists (GB, LP, DR, SS and RC), not aware of the clinical and prognostic data of the corresponding patients, evaluated all histological specimens separately. Discrepancies in the evaluation were resolved by consensus.

The cohort of cases included 39 UMs with metastasis and 46 non metastatic UMs. We collected the following clinical data: tumor size and location, determined with standard methods, such as ophthalmoscopy and A and B scan ultrasonography, and onset of metastases, evaluated with physical examination, liver ultrasound and total body computed tomography (CT). The A-scan ultrasound is a mono-dimensional amplitude modulation scan, mainly used in ophthalmology to study routine visual disorders and/or to investigate the size and ultrasound features of intraocular masses; conversely, B-scan ultrasound refers to a two-dimensional, cross-section brightness scan, that, if combined to A-scan imaging, allows to get more details about the tumor, including exact anatomic site, borders, shape, and size, and to obtain a more detailed preoperative diagnosis.

### Evaluation of Blood Vascular Microvessel Density (MVD)

MVD was evaluated by five pathologists (GB, LP, DR, SS and RC), who identified vascular hotspots on immunohistochemical sections of the UM cohort stained for anti-CD31 antibody by a light microscope at 4× and 10× magnifications, as previously described (46). MVD consisted of the amount of vessels per mm^2^ (conversion factor: 1 mm^2^ = 4 high power fields (HPFs) at 40× magnification inside vascular hotspots. Areas with ≥50 of viable tumor cells were considered usable for counting; Presence of diffuse necrotic, hemorrhagic and totally pigmented areas were considered as exclusion factors. Each CD31+ endothelial cell and/or lumen for long branched vessels were counted. Positive staining of small clusters of ≥2 endothelial cells was assessed as one single vascular structure. MVD levels were considered high if > the median value, low if < the median value.

### Immunohistochemistry

Sections were treated for immunohistochemical analyses with standard methods (47), using the streptavidin/biotin-based system for immunoperoxidase; briefly, after appropriate deparaffinization and pre-treatments, sections were incubated for 30 min at 37°C with primary polyclonal rabbit anti-human antisera against Beclin-1 (working dilution 1:250; Abcam, Cambridge, MA, USA), p62 (working dilution 1:200; Abcam, Cambridge, MA, USA), ATG7 (working dilution 1:100; Abcam, San Francisco, CA, USA) and with primary monoclonal mouse anti-human antiserum against CD31 (JC70A; working dilution 1:40; DAKO, Glostrup, Denmark). The secondary biotinylated antibody was applied for 30 min at room temperature, followed by the streptavidin–biotin–peroxidase complex for a further 30 min at room temperature. The immunoreaction was visualized by incubating the sections for 4 min in a 0.1% 3,3’-diaminobenzidine (DAB) and 0.02% hydrogen peroxide solution (DAB substrate kit, Vector Laboratories, CA, USA).

### Evaluation of Immunohistochemistry

p62 and Beclin-1 were immunoexpressed both in the cytoplasm and in the nucleus of neoplastic cells, while ATG-7 only showed cytoplasmic staining. Negative controls were additionally used by omitting the primary antibody. Intensity of staining (IS) was scored on a scale of 0–3 (47), as follows: absence of staining = 0, weak staining = 1, moderate staining = 2, strong staining = 3. The percentage of immunopositive cells (Extent Score, ES) was graded by five groups (47): <5% (0); 5–30% (+); 31–50% (++); 51–75% (+++), and >75% (++++). Counting was performed at 200× magnification. The intensity reactivity score (IRS) was obtained by multiplying IS and ES (47): IRS < 6 was interpreted as low expression (L-IRS), IRS > 6 as high expression (H-IRS).

### Statistical Analysis

The rates of high and low levels of beclin-1, p62, ATG7 expression in melanoma of patients with and without metastasis were non parametrically compared by chi-square test. Agreement among observers was tested by Cohen K.

Univariate and multivariate analyses, based on a Cox proportional hazards regression model (time free from metastasis as outcome), were performed; gender, age, melanoma location (choroid or ciliary body), temporal or nasal location, cells type (epithelioid, spindle cells or mixed), echographic parameters (height, greatest diameter), and expression level (low and high) of beclin-1, p62, ATG7 and MVD were all included in this model. If a predictor had a P value <0.15 (cut off) in the univariate analysis, it was included in the multivariate one. Survival analysis according to beclin-1, p62, ATG7 and MVD expression levels (high and low) was performed by Kaplan-Meyer test; survival rates were compared by log-rank (Mantel-Cox) test. P values < 0.05 were considered as statistically significant.

## Results

### Clinico-Pathological Features of Uveal Melanomas

Eighty-five patients were part of the study (44 males; 41 females); median age was 67 years (range 29–85). Choroid was the only affected site in 64 cases, while choroid and ciliary body were simultaneously involved in 21 cases. Extrascleral extension was identified in 3 cases. Histologically, 20 cases were diagnosed as epithelioid cell, 25 as spindle cell, while 40 cases as mixed epithelioid and spindle cell UMs. According to the “TNM classification of malignant tumors”, pathological T stage was: pT1a in 15 patients, pT1b in 4 patients, pT2a in 45 patients, pT2b in 16 patients, pT2d in 1 patient, pT3a in 21 patients, pT3b in 10 patients, pT3d in 1 patient, pT4a in 8 patients, pT4b in 8 patients and pT4d in 1 patient. Liver metastases were present in 39 patients. Median follow-up period was 58 months (range 8–138).

**Figure 1:**
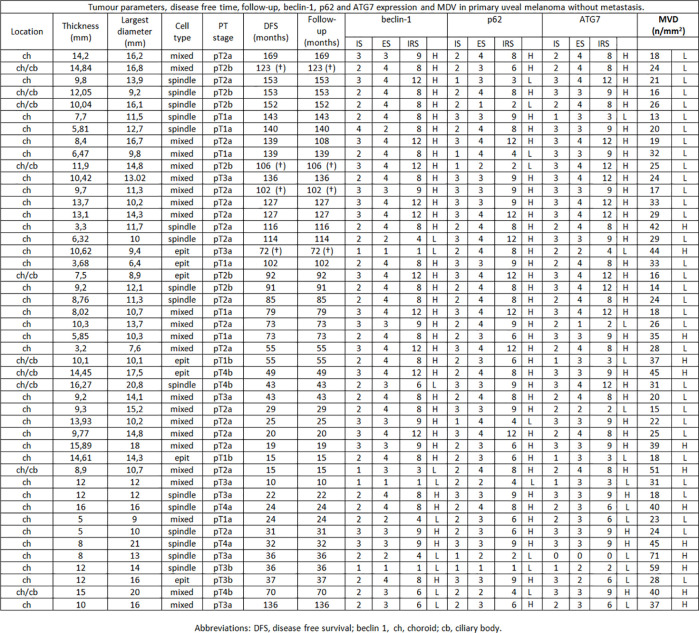
Tumor parameters, disease free time, follow-up, beclin-1, p62 and ATG7 expression and MVD in primary uveal melanoma without metastasis.

**Figure 2:**
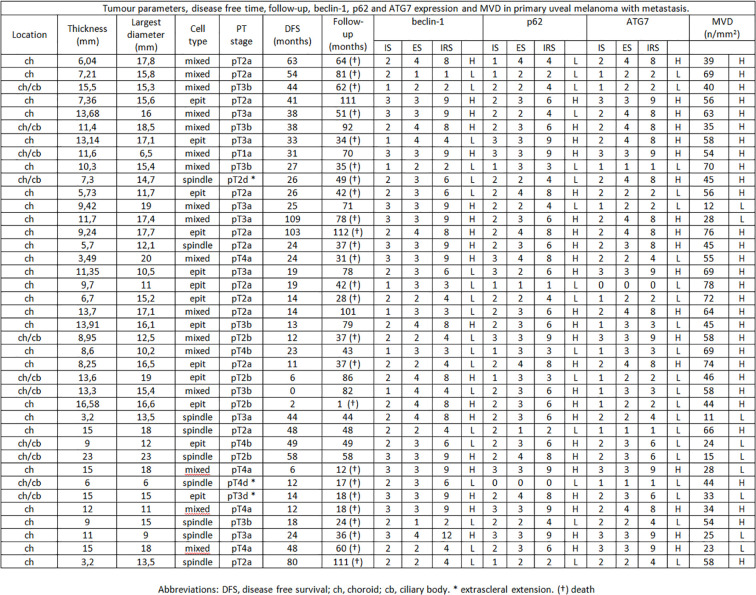
Tumor parameters, disease free time, follow-up, beclin-1, p62 and ATG7 expression and MVD in primary uveal melanoma with metastasis.

**Figure 3:**
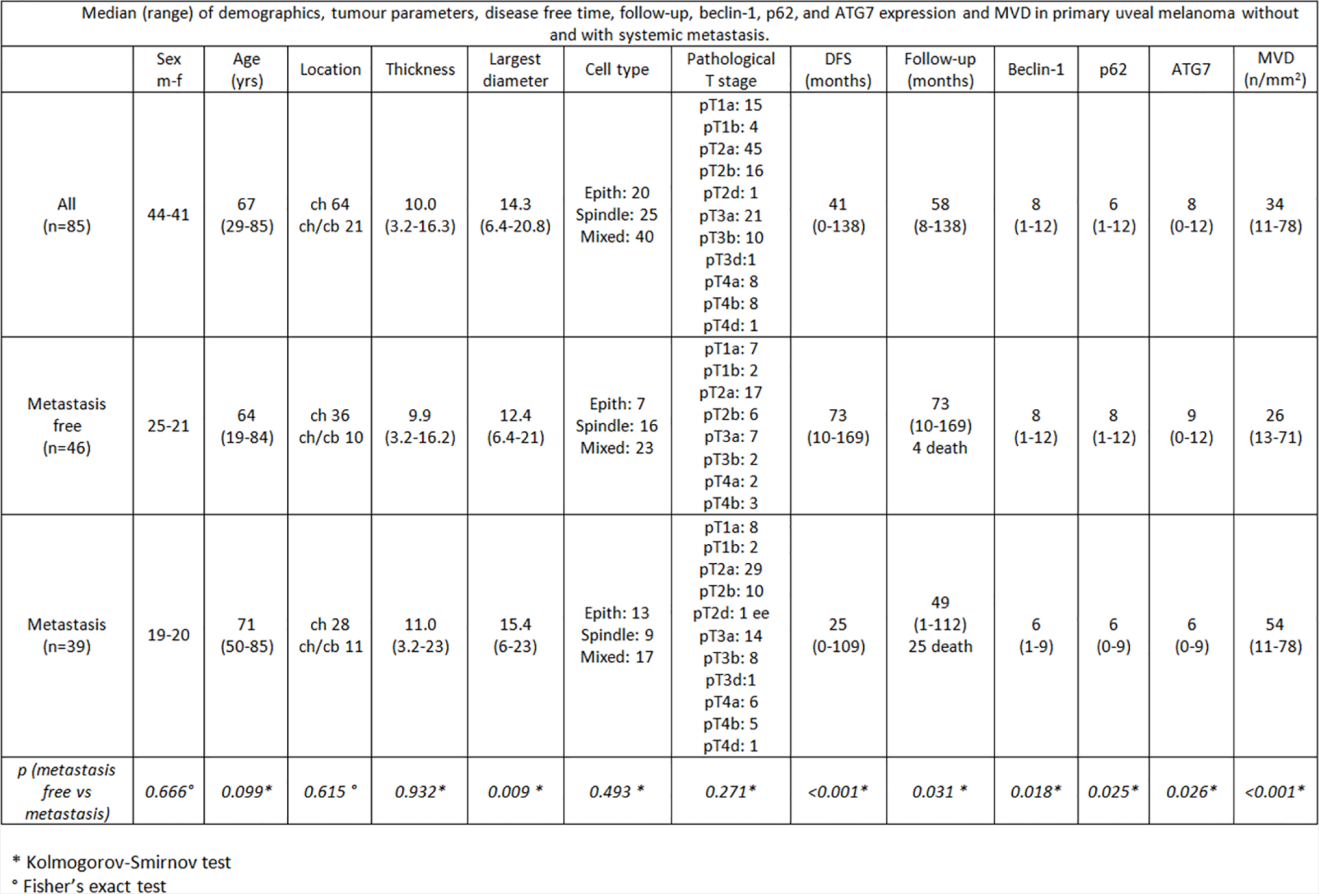
Median (range) of demographics, tumor parameters, disease free time, follow-up, beclin-1, p62, and ATG7 expression and MVD in primary uveal melanoma without and with systemic metastasis.

Among the 46 non metastatic patients, 25 were males and 21 females; median age was 64 years (range 19–84). Out of 39 metastatic patients, 19 were males and 20 females; median age was 71 (range 50–85). 25/39 patients with metastases died during the follow-up time for disease progression (**Figures 1**, **2**). No significant difference was seen in median age, location of the melanoma (choroid or choroid/ciliary body), tumor thickness, histological type, extrascleral invasion and pathological T stage between metastatic and non metastatic patients; UMs with greater median largest diameter (15.4 mm vs 12.4 mm, p = 0.009), lower median Beclin-1 expression (6 vs 8, p = 0.018), lower median p62 expression (6 vs 8, p = 0.025), lower median ATG7 expression (6 vs 9, p = 0.026) and higher median MVD levels (54 vs 26, p < 0.001) were observed in metastatic patients, who also presented lower median disease free survival (25 months vs 73 months, p < 0.001) (**Figure 3**).

### Immunohistochemical Expression of Autophagy-Related Proteins and Correlation With Clinico-Pathological Factors and MVD in Uveal Melanomas

**Figure 4:**
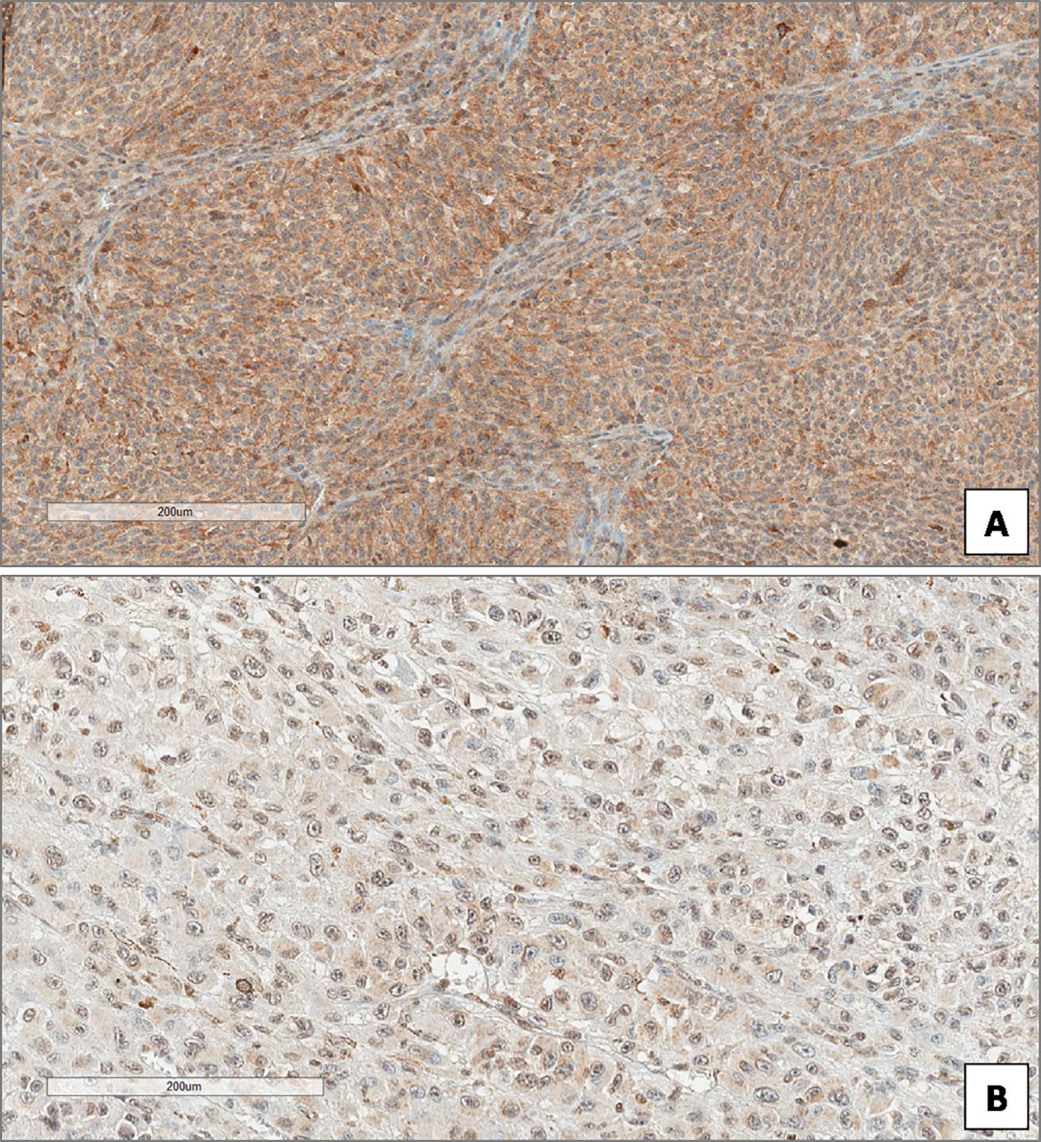
High **(A)** and low **(B)** expression of Beclin-1 in uveal melanoma. (Immunoperoxidase stain; original magnifications 100×).

**Figure 5:**
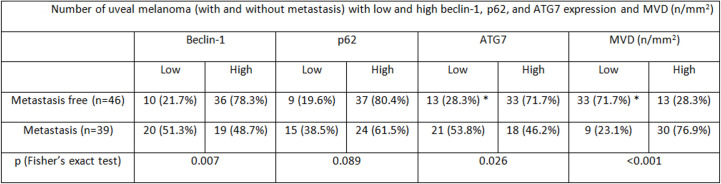
Number of uveal melanoma (with and without metastasis) with low and high beclin-1, p62, and ATG7 expression and MVD (n/mm^2^).

In the overall cohort of patients included in the study (n = 85) the median Beclin-1 value was 8. Beclin-1 expression was high in 55 Iand low in 20 UMs (**Figures 4A, B** **)**. Among 46 primary non metastatic UMs, only 10/46 cases (21.7%) showed L-IRS, while the other 36 UMs showed H-IRS (78.3%) (Fisher’s exact test, p = 0.007, **Figure 5**). In 39 primary metastatic UMs 19/39 cases (48.7%) had H-IRS, while L-IRS was found in 20/39 UMs (51.3%) (Fisher’s exact test, p = 0.007, **Figure 5**).

**Figure 6:**
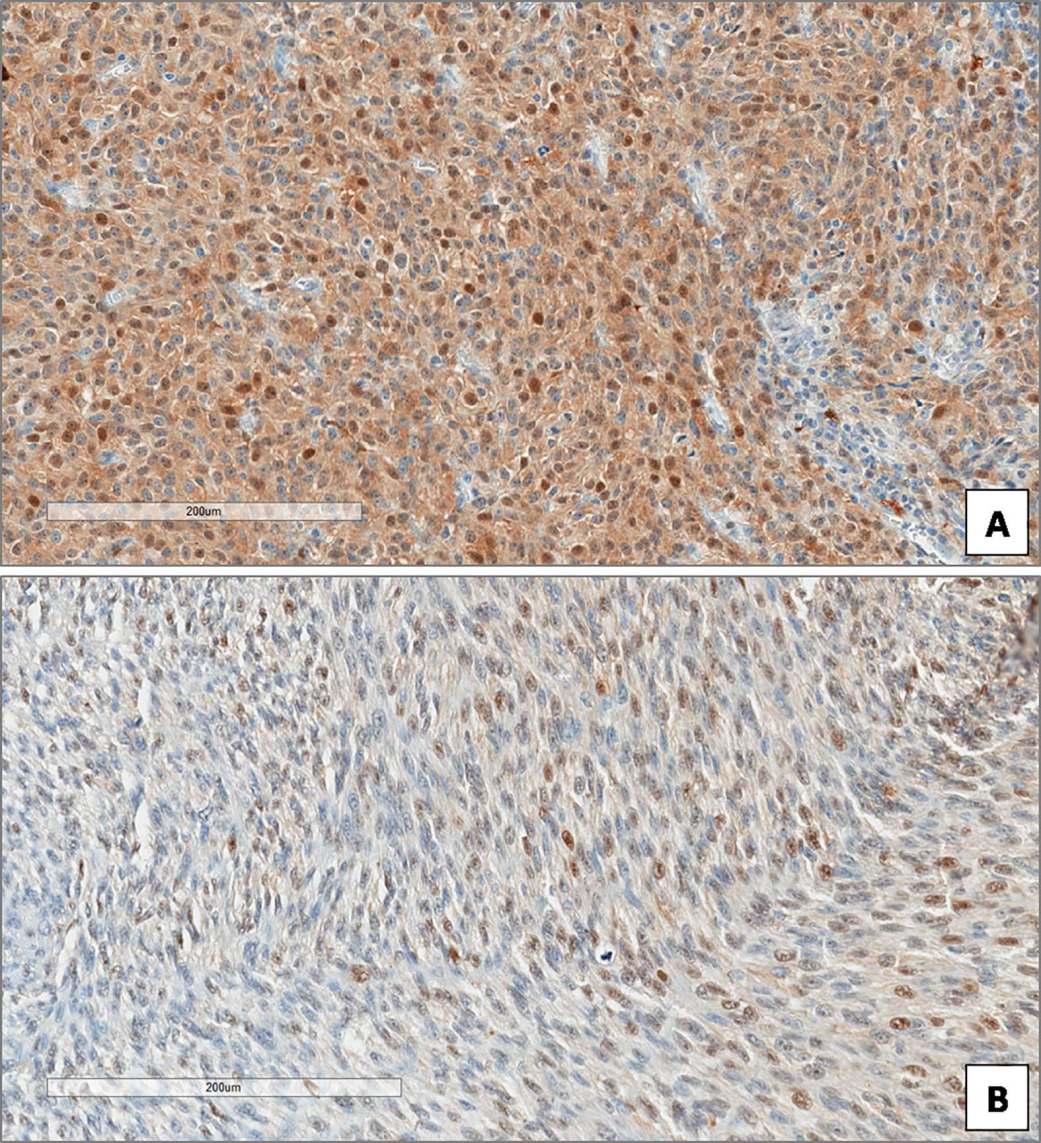
High **(A)** and low **(B)** expression of p62 in uveal melanoma. (Immunoperoxidase stain; original magnifications 100×).

Among the 85 patients studied, the median p62 value was 6. p62 levels were high in 61 and low in 24 cases (**Figures 6A, B** **)**. Among metastasis-free patients, only 9/46 cases (19.6%) showed L-IRS, while H-IRS was observed in the remaining 37/46 cases (80.4%) (Fisher’s exact test, p = 0.089, **Figure 5**). 15/39 metastatic patients (38.5%) showed L-IRS, while 24/39 (61.5%) had H-IRS (Fisher’s exact test, p = 0.089, **Figure 5**).

**Figure 7:**
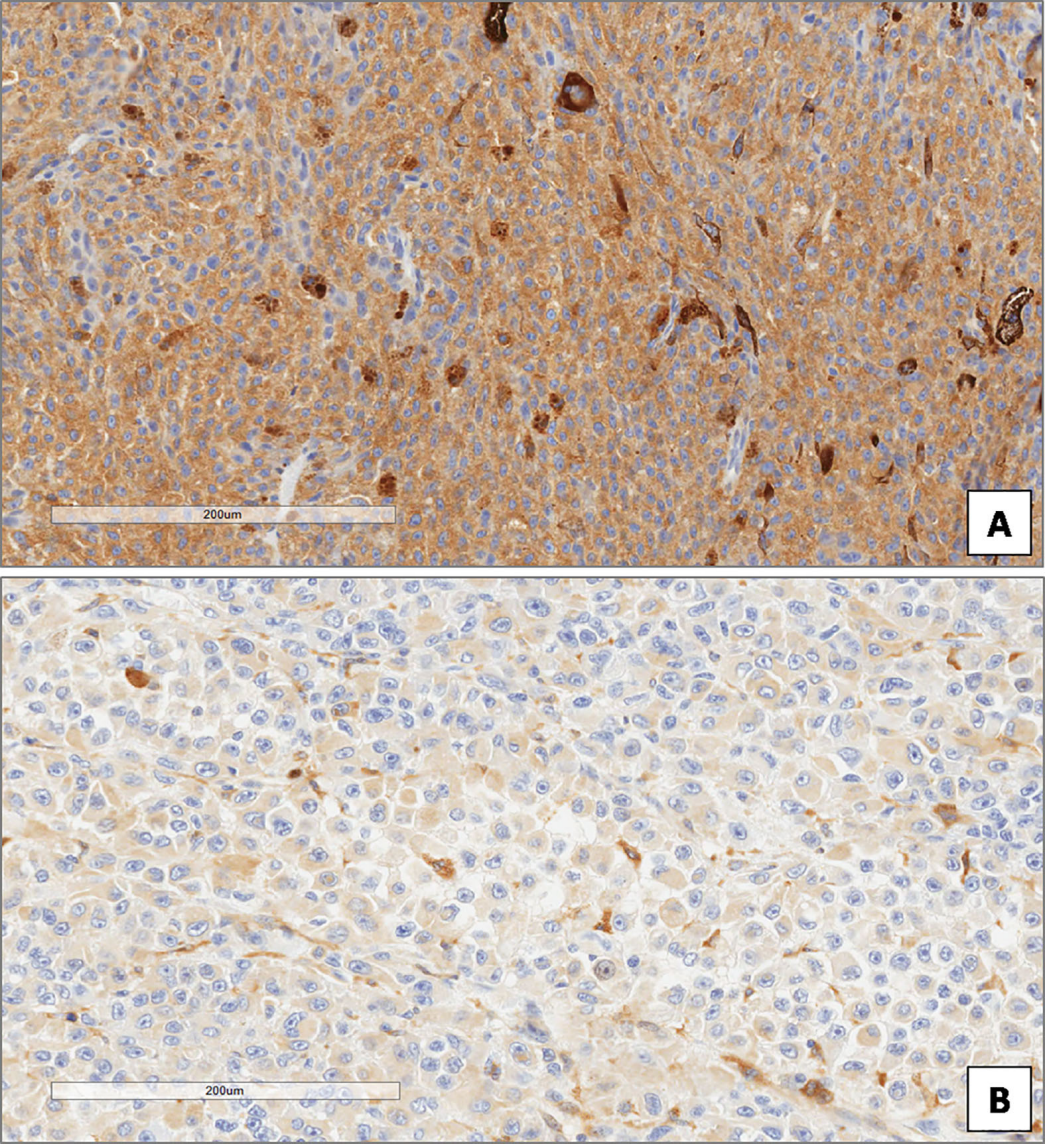
High **(A)** and low **(B)** expression of ATG7 in uveal melanoma. (Immunoperoxidase stain; original magnifications 100×).

In the whole group of UMs, the median ATG7 value was 8. ATG7 immunoexpression was high in 51 patients and low in 34 (**Figures 7A, B** **)**. Out of the 46 patients without metastatic disease, ATG7 L-IRS was found only in 13/46 non metastatic UMs (28.3%), while 33/46 (71.7%) had ATG7 H-IRS (Fisher’s exact test, p = 0.026, **Figure 5**). 21/39 metastatic patients (53.8%) showed ATG7 L-IRS, while the remaining 18/39 (46.2%) ATG7 H-IRS (Fisher’s exact test, p = 0.026, **Figure 5**).

Out of 85 patients studied, the median MVD value was 34 (range 11–78). MVD levels were high in 43 and low in 42 cases. Among metastasis-free patients, MVD was low in 33/46 (71.7%), while high in the remaining 13/46 cases (28.3%) (Fisher’s exact test, p < 0.001, **Figure 5**). 30/39 metastatic patients (76.9%) showed high MVD, while only 9/39 (23.1%) showed low MVD (Fisher’s exact test, p < 0.001, **Figure 5**).

Factors related to the presence of metastasis at univariate analysis on a Cox proportional hazards regression model were: age (p=0.011), diameter (p=0.044), epithelioid cell type (p=0.017), pT stage (p=0.023), and beclin-1 level (p=0.001), p62 level (p=0.079), ATG7 (p=0.002) and MVD (p<0.001).

At multivariate analysis MVD (p=0.009), epithelioid cell type (p=0.014), diameter (0.026) and beclin-1 level (p=0.035) were significant.

No correlation was found between histological type and beclin-1 expression (Spearman’s rho p=0.289), p62 expression (Spearman’s rho p=0.568), ATG7 (Spearman’s rho p=0.127), and MVD (Spearman’s rho p=0.087).

**Figure 8:**
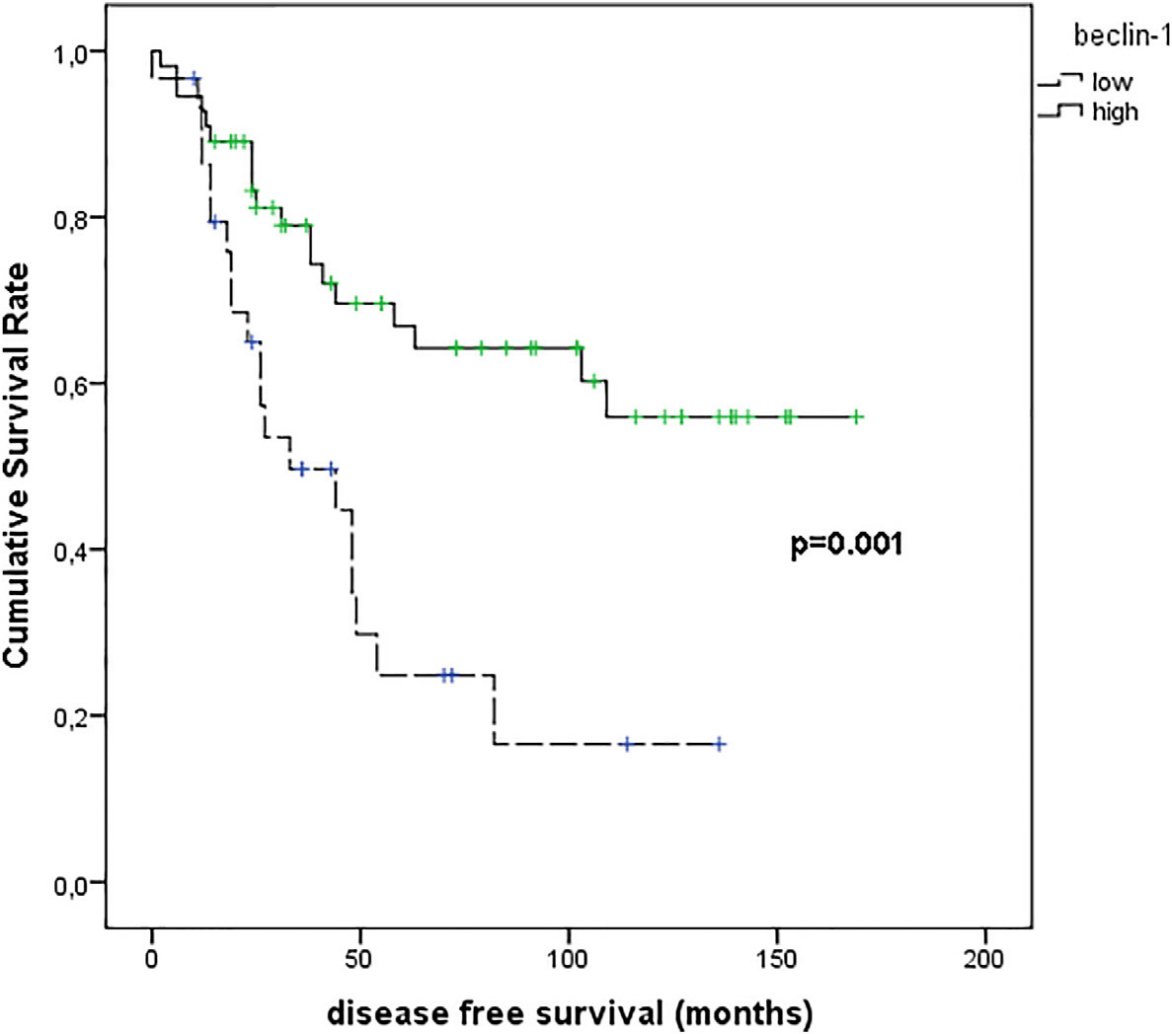
Kaplan–Meier survival analyses in patients with uveal melanomas with low and high Beclin-1 expression.

**Figure 8** shows the results of Kaplan–Meier survival analyses in patients with uveal melanomas with low and high beclin-1 expression. The mean survival time free from metastasis (SE, with 95% CI) estimated were respectively: 50.7 (9.1) (CI: 32.9 to 68.5) and 113.9 (9.9) (CI: 94.5 to 133.4). The log-rank test showed a significant difference (p=0.001) between the two groups.

**Figure 9:**
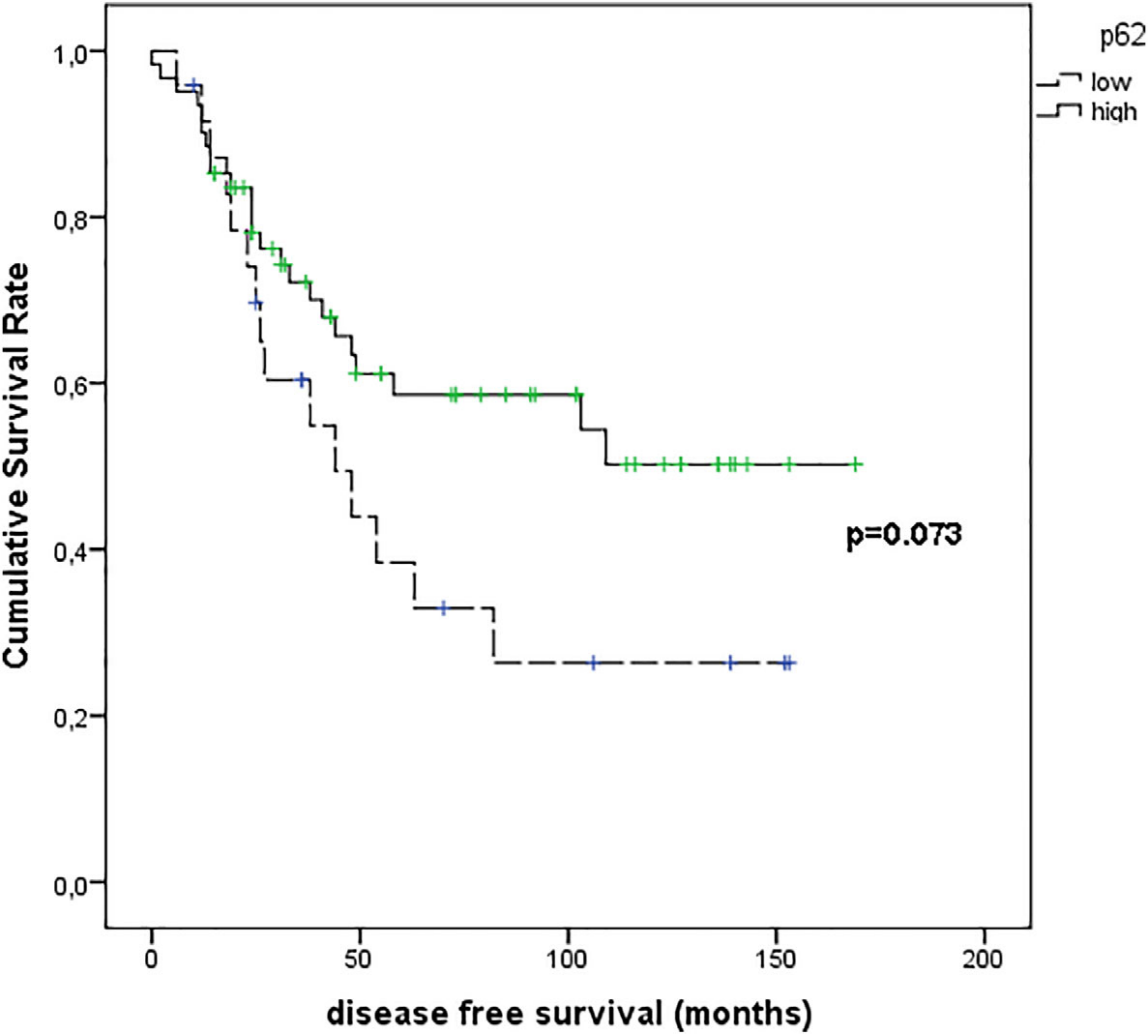
Kaplan–Meier survival analyses in patients with uveal melanomas with low and high p62 expression.

**Figure 9** shows the results of Kaplan–Meier survival analyses in patients with uveal melanomas with low and high p62 expression. The mean survival time free from metastasis (SE, with 95% CI) estimated were respectively: 66.8 (12.3) (CI: 42.7 to 90.9) and 104.9 (9.8) (CI: 85.6 to 124.1). The log-rank test showed no significant difference (p=0.073) between the two groups.

**Figure 10:**
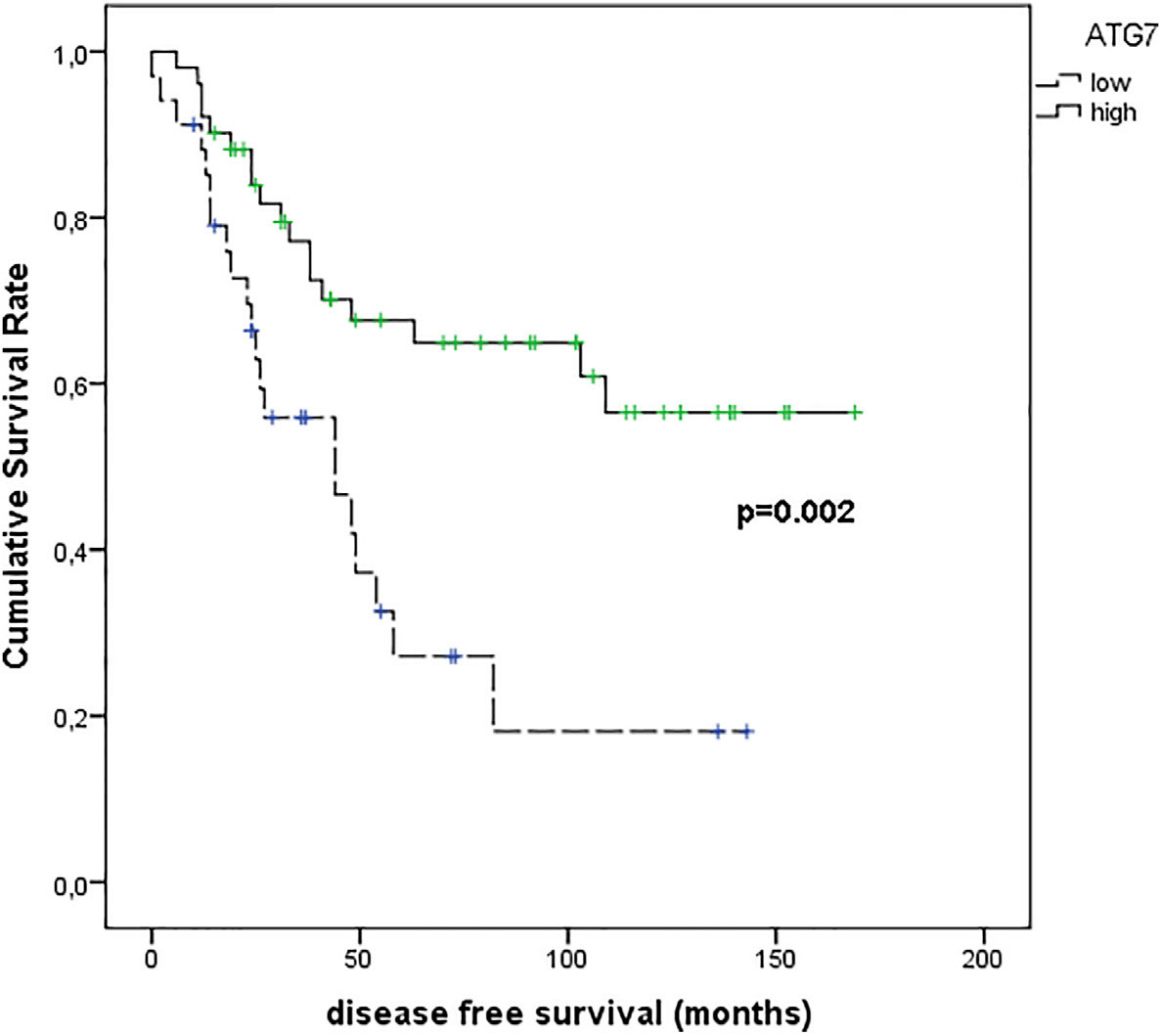
Kaplan–Meier survival analyses in patients with uveal melanomas with low and high ATG7 expression.

**Figure 10** shows the results of Kaplan–Meier survival analyses in patients with uveal melanomas with low and high ATG7 expression. The mean survival time free from metastasis (SE, with 95% CI) estimated were respectively: 54.8 (9.7) (CI: 35.8 to 73.9) and 114.6 (10.1) (CI: 94.9 to 134.3). The log-rank test showed a significant difference (p=0.002) between the two groups.

**Figure 11:**
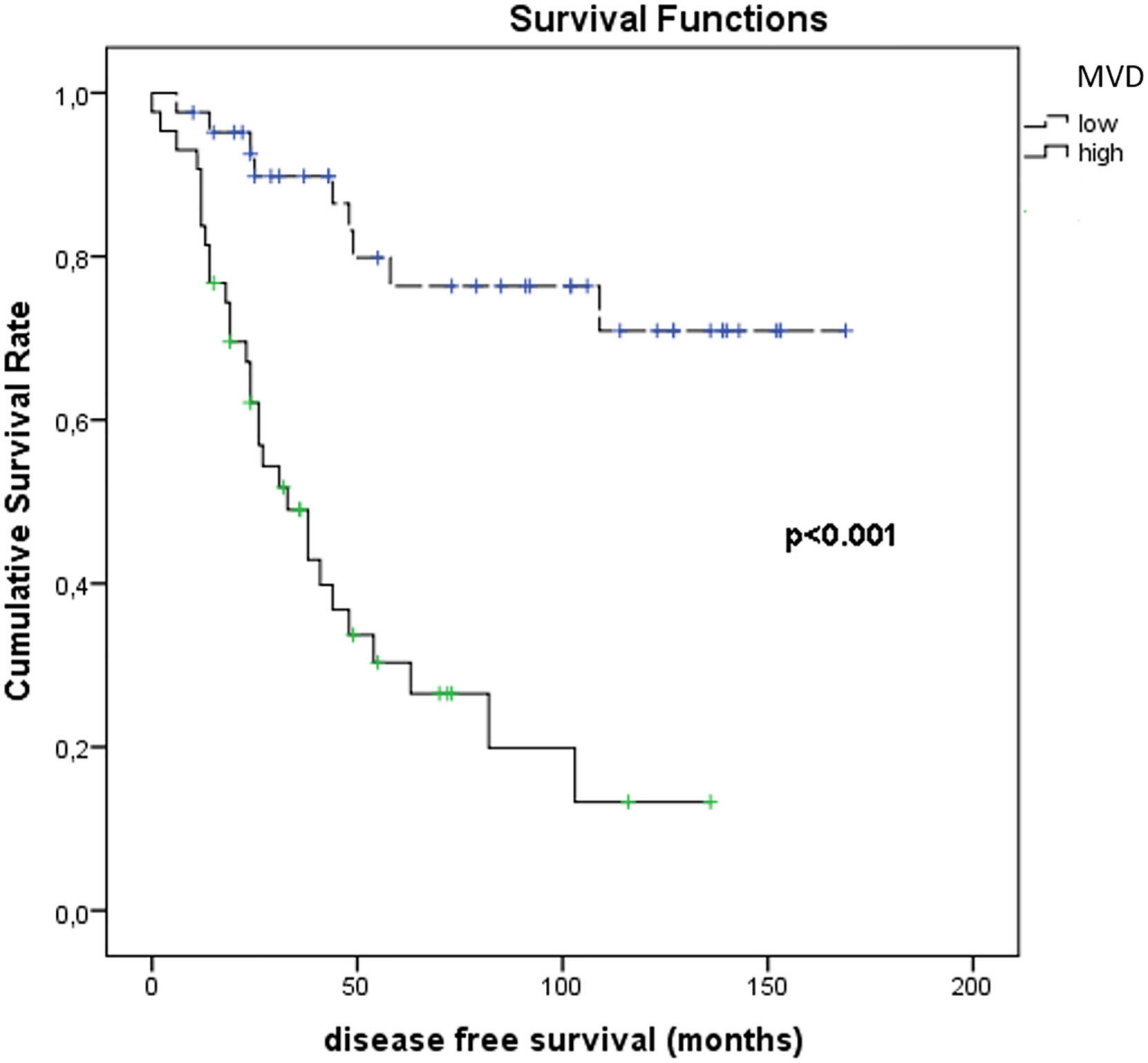
Kaplan–Meier survival analyses in patients with uveal melanomas with low and high MVD count.

**Figure 11** shows the results of Kaplan–Meier survival analyses in patients with uveal melanomas with low and high MVD expression. The mean survival time free from metastasis (SE, with 95% CI) estimated were respectively: 50.0 (7.4) (CI: 35.5 to 64.6) and 134.3 (10.0) (CI: 114.7 to 153.9). The log-rank test showed a significant difference (p<0.001) between the two groups.

**Figure 12:**
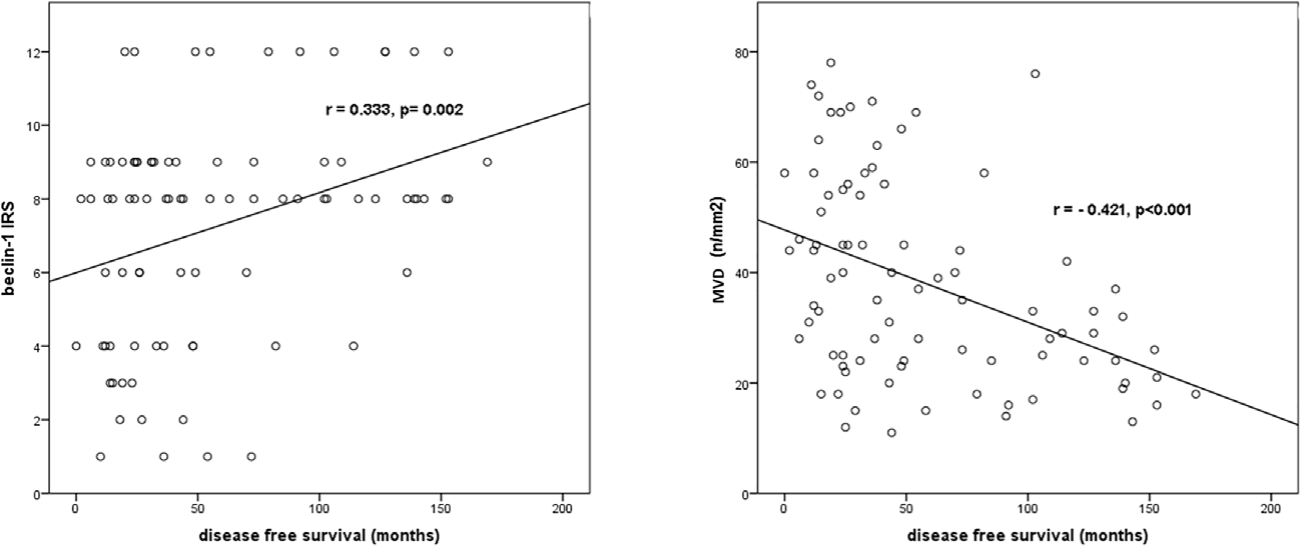
Correlation analysis between beclin-1 IRS, MVD values and DFS.

The results of the correlation analysis between Beclin-1 IRS, MVD values and DFS are displayed in **Figure 12**.

## Discussion

Due to its apparent “indolence” and slow progression, UM may be considered as a tumor with ambiguous clinical course, marked by the occurrence of liver metastases in almost all cases even after 10–15 years after the first diagnosis. Several clinical and histopathological factors have been traditionally included among the ones negatively affecting the prognosis of UM: advanced age and stage at diagnosis, male gender, tumor thickness and largest diameter, ocular/cutaneous melanocytosis, localization to ciliary bodies, extrascleral invasion. By a morphological microscopic point of view, the prevalence of an epithelioid cytotype (48), high mitotic index and tumor associated angiogenesis (expressed as MVD), necrosis, are considered factors linked to a more aggressive tumor behaviour. As well, tumor infiltrating lymphocytes and/or histiocytes (49) and cytogenetic factors including monosomy 3, chromosome 8q-gain or 8p-loss, chromosome 1p-loss, chromosome 6q-loss, and high expression of insulin-like growth factor-1 receptor (IGF-1R) (50), also contribute to the definition of the class risk of each case of UM. In particular, the reduction/loss of the nuclear immunostaining for the BRCA1 associated protein-1 (BAP-1), reflecting the presence of inactivating mutations of the corresponding gene, represents a poor prognostic factor for of UM (51, 52).

We previously identified the immunohistochemical expression of proteins, such as RKIP, pRKIP, ADAM10, ABCB5, SPANX-C, and MacroH2A, as possible useful prognostic markers in UM (47, 53–56). However, despite the efforts of the scientific community and the increase of possible new biomarkers, the response to therapy of patients with advanced disease are yet dismal, so the identification of new prognostic and predictive tissue markers for UM is still an urgent need.

Autophagy looks to act as “double-edged sword” in cancers (57), either contributing to the induction of apoptosis in aberrantly proliferating or damaged cells in early tumor stages, or contributing to the survival of advanced cancer cells upon hostile events, as hypoxia and reduced availability of nutrients, or antineoplastic drugs.

Beclin-1, has a central role in the autophagic process as a major member of the macro-autophagic phase, and ranks above the most studied proteins. A great variability in the expression of this macroautophagy-related protein in human tumor tissues has been documented, sometimes with frankly divergent results. The loss/reduction of Beclin-1 expression has been shown to be closely related either to a better survival, as in endometrial, renal, gastric and colorectal cancers (29, 58–60), or to a reduced disease-free survival (DFS) and poorer prognosis in ovarian cancer patients receiving combined therapy with platinum and taxanes (61), and in a cohort of non-small cell lung cancer (NSCLC), in which the expression of the protein showed an independent positive prognostic factor (62). In squamous cell carcinomas of the uterine cervix the overexpression of Beclin-1 has been reported to inhibit metastatic progression *in vitro* (63), and in non-Hodgkin lymphomas (NHL), the increased expression of Beclin-1 has been found related to an increase in LC3-positive autophagic vacuoles and a better outcome of patients after chemotherapy (64). In melanocytic skin lesions, a gradual decrease of cytoplasmic expression of Beclin-1 has been found correlated with the progressive gain and increase of malignancy (65). The immunohistochemical expression of Beclin-1 has been detected, in fact, in about 100% of benign nevi and 86.4% of dysplastic nevi, decreasing to 54.3% in primary melanomas and up to 26.7% in melanoma metastases. Further studies showed instead that vemurafenib reduced miR-216b level resulting in upregulation of Beclin-1 (66), has been reported as a mechanism of drug-induced autophagy, allowing vemurafenib-resistant melanoma cells to employ autophagosomes to secret ATP to enhance cell migration and invasion (67).

The prognostic role of autophagy and Beclin-1 in UM has been investigated by Giatromanolaki et al. (68), who found either the overexpression and the reduced expression of BECN1 associated with poor prognosis.

Our results indicated a prognostic role of Beclin-1 in UM, with a lower risk of metastasis and higher disease-free survival times observed in UM cases with higher immunohistochemical expression of the protein. By converse, our population of UM with low expression of Beclin-1 was characterized by a higher metastatic risk. In our study, no statistically significative difference in the immunohistochemical expression of ATG7 and p62 proteins between metastasizing and non-metastasizing primary UM was detected. In our series of cases, the expression of these two macro-autophagy related proteins didn’t show any predictive value for metastatic risk of UM. Among the autophagy-related proteins analyzed in our study, then, only Beclin-1 resulted promising as a new possible immunohistochemical marker able to predict metastatic risk in patients with primary UM.

Some considerations have to be made, before any conclusion at this regard may be got. To date, there is still the lack of robust markers, improved staining protocols, and standardized interpretation to measure autophagy activity in archived tissue, and we must interpret our data with caution, strictly integrating them with clinical and follow-up data (22).

New specific markers identifying the core autophagy components and its upstream and downstream regulators, and the identification of specific gene “signatures” are needed for a more accurate assessment of the autophagic activity in FFPE tumor samples, at diagnostic and/or prognostics level, and to predict response to new autophagy-targeted treatment. Moreover, controversies remain regarding whether to inhibit or enhance autophagy in cancer, considering that, besides activation of endocytosis, the deregulation of autophagy-related genes has been associated also with cell death pathways and DNA repair responses (non-autophagy functions) (69, 70). Nevertheless, our finding of a strict correlation between Beclin-1 expression and the clinical outcome of UM support the idea that alteration in autophagy may be a particularly attracting targetable way to treat UM progression, as it has been proposed for skin melanoma. In cutaneous melanoma, even considering the large patient-to-patient variability of drug resistance mechanisms, non-apoptotic cell death has been considered as alternative therapeutic target when induction of apoptosis is impaired (71).

In addition, the finding of an altered expression of Beclin-1 in our study population sounds particularly attractive, if we consider that recent evidence suggests that alterations in autophagy may be a major mechanism of tumor escape from immune surveillance also by interfering with signaling pathways in tumor and immune cells (72) and autophagy-associated cell death has emerged as a key immunogenic mechanism able to potentiate tumor response to therapy in several human malignancies and in skin melanoma (29, 58–68). In particular, recent studies indicate that targeting autophagy in melanoma cells in combination with immunotherapy could gain results in promoting tumor regression, and autophagy has been shown to act a pivotal role in dendritic cell and T-lymphocyte infiltration (73, 74) in immune-competent animal models. These observations sound particularly interesting, that targeting autophagy could offer a new chance for UM patients non-responder to targeted and immunotherapeutic protocols active on most of human solid malignancies.

Further considerations emerge, in addition, from the analysis of several existing data indicating additional non-autophagic roles of beclin-1 expression in cancer. Besides inducing autophagy, this protein has been documented to be involved in parallel in growth signaling pathways AKT (protein kinase B) and ERK (kinase regulated by extracellular signals). It is therefore plausible that the increased expression of Beclin-1 inhibits these signaling pathways, limiting neoplastic growth (75).

Likewise, interesting consideration could derive from the analysis of the possible correlation between the expression of Beclin-1and uveal melanoma angiogenesis. As before outlined, increased angiogenesis, expressed as micro-vascular density (MVD) evaluated immunohistochemically, has been associated with poor prognosis (high metastasis and mortality rate) in uveal melanoma (76, 77). This finding it is not surprisingly, if we consider that UM metastasizes solely *via* the haematogenous route. In a previous study, we found that the chemokine receptor CXCR4, a prognostic factor in cutaneous melanoma being involved in angiogenesis and metastasis formation, is commonly expressed in uveal melanoma and correlates to the epithelioid-mixed cell type (78). In addition, Brouwer NJ and colleagues analyzed the correlation between several angiogenesis-related cytokines and the development of tumor vessels of primary UM, founding that a high MVD is associated with an increased expression of angiopoietin 2, Von Willebrand Factor, and a decreased expression of vascular endothelial growth factor B (VEGF-B) (79). It has been found that the expression of endothelial growth factors (VEGF, MMP-9) is negatively modulated by high levels of Beclin-1 (80).

Basing on this finding, the results concerning Beclin-1 expression in our study population lead us to hypothesize that a similar scenario could have important effects on tumor angiogenesis in UM. This could at least in part address the many still unanswered questions concerning the complex interplay between the regulation angiogenesis pathways and its correlation with UM biological behavior.

The mechanisms by which Beclin-1 negatively modulates tumor growth are not fully understood. A recent study outlined that Beclin-1 may interact also with members of the bcl-2 protein, acting as a tumor suppressor protein (81), and this finding could explain, at least partially, its positive prognostic role in different types of human cancers. The conflicting data in literature indicate in addition different patterns and roles of Beclin-1 expression depending on neoplastic cell types.

To date, many concerns need to be still overcome, to fully clarify the ultimate role of Beclin-1 expression in cancer progression and cancer response to therapy in UM, and a further study on larger, multi-institutional series of cases is currently in progress to definitively validate our results. Nevertheless, the results of our study indicate that a relationship between the immunohistochemical expression of Beclin-1 and the biological behavior of UM exists, and this could open up new prognostic and therapeutic strategies for this peculiar, deadly malignant tumor.

## Data Availability Statement

The original contributions presented in the study are included in the article/supplementary material. Further inquiries can be directed to the corresponding author.

## Ethics Statement

The studies involving human participants were reviewed and approved by Comitato Etico Catania 1, Azienda Ospedaliero Universitaria Policlinico “G.Rodolico - San Marco” *via* S.Sofia, 78 - 95123 Catania. The patients/participants provided their written informed consent to participate in this study.

## Author Contributions

GB, AI, and AR contributed to implementation of the study and to the writing of the manuscript. SS and RC, as co-senior authors, contributed to the design of the study. GT, DR, AR, AL, MR, and SV were involved in planning and supervised the study. GB, RC, AI, SS, DR, LP, and GT performed the histopathologic diagnoses and the complete analysis of the surgery material. All authors contributed to the article and approved the submitted version.

## Conflict of Interest

The authors declare that the research was conducted in the absence of any commercial or financial relationships that could be construed as a potential conflict of interest.
